# Assessing the effect of *Mentha longifolia* essential oils on COX-2 expression in animal model of sepsis induced by caecal ligation and puncture

**DOI:** 10.1080/13880209.2018.1510972

**Published:** 2018-12-04

**Authors:** Abolfazl Dadkhah, Faezeh Fatemi, Azadeh Rasooli, Mohammad Reza Mohammadi Malayeri, Fatemeh Torabi

**Affiliations:** a Department of Medicine, Faculty of Medicine, Qom Branch, Islamic Azad University, Qom, Iran;; b Materials and Nuclear Fuel Research School, Nuclear Science and Technology Research Institute, Tehran, Iran;; c Department of Biochemistry, Faculty of Science, Payame-e-Noor University, Tehran, Iran;; d Department of Pathobiology, Faculty of Veterinary Medicine, Garmsar Branch, Islamic Azad University, Garmsar, Iran;; e Department of Physiology, Faculty of Science, Qom Branch, Islamic Azad University, Qom, Iran

**Keywords:** Oxidative stress, antioxidant activity, anti-inflammatory activity, hepatoprotection, LP, GSH, FRAP

## Abstract

**Context:***Mentha longifolia* L. (Lamiaceae), a traditional Iranian plant, possesses antimicrobial and antioxidant activities.

**Objective:** We investigated the potential protective effects of *M. longifolia* essential oils (E.Os) on caecal ligation and puncture (CLP) induced liver injury.

**Materials and methods:** Wistar Albino rats (*n* = 50) were grouped as follows: (1) a laparotomy group (LAP); (2) a CLP group (CLP); (3) the treatment groups received orally the E.Os (50 and 100 mg/kg b.w) and indomethacin (2 mg/kg b.w) for 2 weeks. The oxidative stress parameters, liver enzymes and prostaglandin E2 (PGE2) level were measured in liver and plasma tissues. The liver was also harvested for the real time PCR of cyclooxygenase (COX-2) expression following histopathological examinations.

**Results:** The results indicated that the CLP operation significantly increased lipid peroxidation (LP) [1.79-fold], myeloperoxidase (MPO) [2.76-fold], PGE2 [1.56-fold] besides plasma aspartate aminotransferase (AST) [2.4-fold] and alanine aminotransferase (ALT) activities [2.22-fold], while, markedly reduced glutathione (GSH) [0.63-fold] and ferric reducing ability of plasma (FRAP) levels [0.63-fold]. Even COX2 expression significantly increased in the CLP group as compared to the LAP group. Treatments of rats with the E.Os could return all the hepatic and plasma biomarkers to the normal levels. These results were further confirmed by pathological examination on liver indicating that E.Os could successfully improve the CLP-induced liver injuries.

**Discussion and conclusions:** Our findings suggest that E.Os is able to protect liver injuries against sepsis *via* modulating the oxidative stress parameters concomitant with the suppression of inflammatory reactions such as PGE2 and COX-2.

## Introduction

Sepsis refers to a complexity of traumatic injury that develops in one-half of all patients suffering from trauma (Vincent 2000; Angus et al. 2001; Singer et al. 2016). Moreover, in the early stages of sepsis acute events may provoke long-term outcomes such as pulmonary complications and persistent immunosuppression (Reddy et al. 2001; Benjamim et al. 2004), and causes late mortality in 25% of patients who survive severe sepsis (Benjamim et al. 2004). A severe sepsis is caused *via* release of different inflammatory mediators which often induce deleterious effects in the hosts. The harmonized expression of anti-inflammatory molecules regulates the release of inflammatory mediators (Reddy et al. 2001; Marshall 2012). Sepsis is a prevalent complexity that creates extreme oxygen-free radicals. The produced radicals in turn lead to oxidative stress causing multi-organ failure (Peralta et al. 1993). Immune system attacks the body’s own tissues and organs due to bacterial toxins that eventually cause a severe sepsis. Nevertheless, in the condition wherein various organs are affected, it can even lead to death (Hubbard et al. 2005). Any harmful event that causes harm to tissues such as trauma, infection or anoxia eventually leads to cytokine secretion. Inflammatory cells activated through these cytokines abundantly release the toxic oxidizing reactive oxygen species (ROS). Subsequently, this event causes cellular injury through different mechanisms involving increase of myleoperoxidase (MPO) activity, the peroxidation of membrane lipids and reducing the glutathione (GSH) levels (Hubbard et al. 2005; Fatemi et al. 2010). Moreover, COX is the key enzyme for prostaglandin biosynthesis, and inhibition of PG synthesis is at the centre of current anti-inflammatory therapies. There are few evidences indicating that COX-2 is involved in many inflammatory processes and induced in various carcinomas, which suggests that COX-2 plays a key role in inflammation and tumorgenesis (Mutoh et al. 2006; Fujimura et al. 2007). Several reports indicate that herbal and antioxidant drugs induce their antioxidant characteristic *via* being oxygen-free radical’s scavenger (Huh et al. 1994; Gomez-Zubeldia et al. 2000).

*Mentha* [Labiatae (Lamiacea)] species are commonly known as nana in Persian and generally used as a flavouring agent, herbal tea and medicinal plant. *Mentha* is safe and efficient therapeutic agent in hypertension, diabetes and inflammation through various mechanisms (Mokaberinejad et al. 2012). *Mentha longifolia* L. (ML) is native to Southeast Asia. It is commonly termed as wild mint, habak or hasawy. *Mentha longifolia* is an aromatic perennial herb growing at a rapid rate and broadly applied in traditional and herbal medicine as a remedy for many diseases such as gastrointestinal diseases, colds, coughs, influenza, swollen glands and wounds. The aforesaid plant is also considered to be efficacious and profitable for immune system and fighting over secondary infections (Asekun et al. 2007; Murad et al. 2016). Moreover, herbs and spice essential oils considered to be one of the most selections for antimicrobial feed additives. They also demonstrate antifungal, antioxidant, enzymatic and digestion-stimulating activities (Ghaly et al. 2017). Furthermore, the essential oils of *M. longifolia* exhibited decongestant, antispasmodic, antioxidant and antimicrobial properties (Hutchings and van Staden 1994; Iqbal et al. 2013).

The conventional inflammation therapies *viz*. non-steroidal anti-inflammatory drugs (NSAIDs), have a significant role to control pain as well as inflammatory conditions (Davies et al. 2000), though with rather discouraging profile of side effects (James and Hawkey 2003). Additionally, few researchers have indicated that oxidative mechanisms are at the origin of inflammation thereby suggesting the antioxidant substances as effective drugs (Habashy et al. 2005; Piechota-Polanczyk and Fichna 2014). These studies revealed the need for new and safe anti-inflammatory drugs to control the sepsis. Besides, there is no study on the role of *M. longifolia* oils as Iranian native plant in treatment of liver disorders. Therefore, the aim of this study was to evaluate the protective effects of *M. longifolia* essential oils on the sepsis-induced liver injury by rat caecal ligation and puncture (CLP) model through the essential oxidative/antioxidant parameters and gene expression of COX-2.

## Materials and methods

### Preparation of *Mentha longifolia* essential oils

*Mentha longifolia* essential oils were prepared from Barij Essence Pharmaceutical Co, Kashan Iran. A voucher specimen (Batch No.:3138-031-93/8 (707051); Sample Serial No.: BE930347) has been deposited at the Barij Essence Company.

### Gas chromatography–mass spectrometry

Analyses were performed using a gas chromatograph of Thermo Finnigan Trace GC (Thermo Electron Co., Waltham, MA) equipped with an AI/AS3000 autosampler, coupled to a mass spectrometer of Thermo Finnigan Automass quadrupole (Thermo Electron). A TR-5 fused-silica column was used with 30 m × 0.25 mm i.d. and 0.25 m film thickness (J&W Scientific, Folsom, CA). The temperature program for the chromatographic run, after optimization, was as follows: initial temperature 50 °C held for 1 min, increased to 280 °C at a rate of 10 C/min, then increased to 280 °C and eventually held for 20 min. Helium (99.999%) was used as carrier gas, at a flow rate of 1.0 mL/min. The injection with the volume of 100 mL/min was set on a split less mode at 280 °C. The MS was operated in electron impact (EI) mode with an ion source temperature of 230 °C. The MS transfer-line temperature was 270 °C. A mass range was recorded in the full-scan mode. Peak identification of objects was based on the retention times and full scan spectra of the standards. The oil components were identified from their GC retention indices, relative to C7–C25 *n*-alkanes, by comparison of their MS spectra with those reported in the literature and by computer matching with the Wiley 5 mass spectra library, whenever possible, by co-injection with standards available in the laboratory (Shibamoto 1987; Davies 1990).

### Determination of antioxidant activity with the 2, 2-diphenyl-1-picrylhydrazyl radical scavenging method

The hydrogen atom or electron donation abilities of the extracts and pure compounds were measured from the bleaching of purple-coloured methanol solution of 2, 2-diphenyl-1-picrylhydrazyl (DPPH). This spectrophotometric assay used the stable radical DPPH as a reagent (Cuendet et al. 1997; Burits and Bucar 2000). We added solutions according to the Table 1. Then, after 30 min of incubation period at room temperature, the absorbance was read against the blank at 517 nm. The inhibitory effects of extracts in percent (*I*%) were calculated by the following formula:

$$I\%=\left(A_{\text{blank}}–\;A_{\text{sample}}/A_{\text{blank}}\right)\times 100.$$

where *A*_blank_ is the absorbance of the control reagent (containing all reagents except the test compound), and *A*_sample_ is the absorbance of the test compound. All the assays were carried out in triplicate.

**Table 1.** DPPH measurement in essential oil.

	Blank	E.O samples	Trolox
Essential oil preparations, 20% v/v (in methanol)	–	50 µl	–
Solution Trolox	–	–	50 µl
0.004% DPPH in methanol	5 mL	5 mL	5 mL
Methanol	50µl	–	–

### Determination of antioxidant activity with the β-carotene-linoleic acid assay

The antioxidant activity of essential oils was determined using the β-carotene bleaching test (Taga et al. 1984). Approximately, 10 mg of β-carotene (type I synthetic) was dissolved in 10 mL of chloroform, and then, 0.2 mL of this solution was added to a boiling flask containing 20 mg linoleic acid and 200 mg Tween 40. Chloroform was removed using a rotary evaporator at 40 °C for 10 min. Then, 50 mL of distilled water saturated with oxygen was added slowly with vigorous agitation to form an emulsion. The emulsion (5 mL) was added to a tube containing 0.2 mL of essential oil solution prepared according to Choi et al. (2000). The absorbance was immediately measured at 470 nm against a blank consisting of an emulsion without β-carotene. The tubes were placed in a water bath at 50 °C and emulsion oxidation was monitored spectrophotometrically by measuring absorbance at 470 nm over a 60 min period. Samples containing 0.2 mL of ethanol instead of essential oils were also monitored and used as a control. Butylated hydroxytoluene (BHT; 1 mM in ethanol), a stable antioxidant, was used as the reference. The antioxidant activity was expressed as inhibition percentage with reference to the control sample after 60 min of incubation, using the following equation:

AA=100(DRC − DRS)/DRC,

Where

AA=antioxidant activity,

DRC=degradation rate of control=[ln  (a/b)/60],

DRS=degradation rate in presence of sample=[ln  (a/b)/60],

a=absorbance at time 0,

b=absorbance at 60min.

### Animal treatments

Male Wistar Albino rats (250 ± 20 g) were purchased from the Pasteur Institute, Iran. Rats were maintained at 23 °C with access to standard food and tap water *ad libitum*. The animals were divided into 5 groups. Laparotomy group received DMSO (essential oil solvent) for 2 weeks, followed by laparatomy. In CLP group, animals received DMSO for 2 weeks followed by CLP operation. In the treatment groups, the essential oils prepared from the plant at 50 and 100 mg/kg b.w were used orally for 2 weeks before CLP operation. Positive control group received indomethacin (2 mg/kg b.w) for 2 weeks before CLP operation. Finally, 24 h after CLP surgery, the heparinated blood samples were collected by heart puncture from all the animals and centrifuged at 3000 *g* for 10 min to obtain the plasma. Liver samples were immediately transferred to ice-cold containers and homogenized (20%, w/v) in the appropriate buffer using a homogenizer. The homogenates were used to measure the biochemical parameters.

### Caecal ligation and puncture model

Sepsis was induced in rat using the CLP method (Hubbard et al. 2005). Briefly, the rats were anesthetized by injection (i.p) of ketamine (90 mg/kg b.w) and xylazine (10 mg/kg b.w) mixture. A small mid abdominal incision (2–3 cm) was made and the caecum was exposed. A distended portion of the caecum just distal to the ileocecal valve was isolated, filled with faecal content and tied with a 3-O silk suture in a manner not to disrupt bowel continuity. The ligated portion of the caecum was punctured twice with a 20-gauge needle. The caecum was then replaced in its original position within the abdomen which then closed with a 3-O suture in two layers. Then, the animals were allowed to recover. In the laparatomy group, the caecum was exposed, manipulated and returned to the peritoneal cavity without being punctured. After surgery, normal saline (3 mL/100 g b.w) was given subcutaneously to all rats to prevent dehydration.

### Ethical approval

This Ethics Committee was based on the World Medical Association Declaration of Helsinki (Adopted by the 18th World Medical Assembly, Helsinki, Finland, June 1964).

### Biochemical analysis

#### Measurement of LP products in liver

A weighed portion of liver tissues was homogenized in phosphate buffer (100 mM, pH 7.0) and used to measure the levels of TBARS as indices for LP. The concentration of TBARS was measured spectrophotometrically using TBA reagent based on the procedure described by Buege and Aust (1978).

#### GSH estimation

GSH was estimated in liver homogenates according to the procedure of Sedlak and Lindsay (1986).

#### Glutathione S-transferase activity

Liver cytosolic glutathione S-transferase (GST) was measured spectrophotometrically using CDNB (a general substrate) according to the procedures described by Habig et al. (1974). Finally, the specific activity was calculated based on the nmol/min/mg protein in samples measured by Bradford assay (Bradford 1976).

#### Ferric reducing ability of plasma assay

This assay was performed using TPTZ reagent as described by Benzie and Strain (1996). This method measures the ability of antioxidants contained in the sample to reduce ferric-tripiridyltriazine (Fe^3+^-TPTZ) to a ferrous form (Fe^2+^) which absorbs light at 593 nm. Ferric reducing ability of plasma (FRAP) level was then calculated by plotting a standard curve of absorbance against μM/L concentration of Fe (II) standard solution.

#### Measurement of myeloperoxidase activity in liver

Tissue myeloperoxidase (MPO) activity was measured, with minor modification, according to the procedure of Hillegass et al. (1990). Weighed tissue samples were homogenized in 50 mM potassium phosphate buffer (pH 6.0), and centrifuged at 41,400 *g* for 10 min. After discarding the supernatant, the pellets were suspended in a solution containing 0.5% hexadecyl-trimethyl-ammonium bromide dissolved in 1 mL potassium phosphate buffer (pH 6.0). After three freeze-thaw cycles, the samples were centrifuged at 41,400 *g* for 10 min. MPO activity was determined by adding 150 μL of the supernatant to 1150 mL of 10 mmol/L phosphate buffer (pH 6.0) and 1 mL of 1.5 mmol/L o-dianisidine hydrochloride containing hydrogen peroxide. The absorbance was measured at 460 nm for 1 min and the rate of change in the absorbance was used to calculate the activities of MPO. MPO activity was expressed as the amount of enzyme that reduces 1 µmol peroxide/min.

#### Prostaglandin estimations

Plasma prostaglandin E2 level was measured using the enzyme-linked Immunosorbent assay kit (ELISA Kit; BioAssay System) according to the producer’s instructions.

#### Liver damage assessment

To assess the hepatocellular injury following CLP, alanine aminotransferase (ALT), aspartate aminotransferase (AST) (Pars Azmoon Co, Iran), alkaline phosphatase (ALP) (Ziest Chem Diagnostics Co, Iran) and total bilirubin (BILI) (Darman Faraz Kave Co, Iran) were measured spectrophotometrically in serum according to the procedure described in the kit purchased.

### Relative qPCR assay

#### Extraction of total RNA & cDNA synthesis

Total RNA from liver tissues was prepared with the RNA total kit (BioBasic Inc, Canada). The extracted total RNA was quantified at OD260 and OD280 with NanoDrop 2000 spectrophotometer (Thermo Scientific). Then, the same amount of total extracted RNA served as the template to synthesize cDNA with PrimeScript ^TM^ RT reagent kit (Takara Bio Inc, Japan) and oligo dt primers (Takara Bio Inc, Japan), according to the manufacturer’s protocol.

#### PCR amplification

Primers for PCR were designed with the Gene Runner software Version 3.05 and primer 3 servers (Table 2). Blast N searches were used to check primer specificity. The cDNA samples were amplified by PCR amplification and then checked by 2.5% agarose gel electrophoresis to ensure whether PCRs contained a product with the expected size.

**Table 2.** Primer sequences.

Primers	Sequence (5′ → 3′)	Product length
COX2 forward	ACCTCTGCGATGCTCTTC	188 bp
COX2 reverse	AGGAATCTCGGCGTAGTAC	
GAPDH forward	TGCCAGCCTCGTCTCATAG	197 bp
GAPDH reverse	ACTGTGCCGTTGAACTTGC	

#### Real-time RT-PCR

The relative-expression of selected gene was carried out with real-time PCR System (Rotor-Gene Q-QIAGEN). The reaction mixture contained 5 μL SYBR Green real-time PCR Master Mix (QIAGEN) which enclosed Taq DNA polymerase, dNTP, MgCl_2_ and SYBR Green I dye, 0.2 μL of a 10 mM solution of sense/anti-sense primer, 0.5 μL of template cDNA added with H_2_O to a total of 10 μL. The negative controls were also designed as above excluded cDNA. Thermal cycling conditions were carried out by an initial denaturation stage at 95 °C for 2 min, followed by 40 cycles at 95 °C for 15 s, 60 °C for 20 s and 72 °C for 20 s. At the completion of each run, melting curves for the amplicons were measured by raising the temperature by 0.3 °C from 57 to 95 °C while monitoring fluorescence. To determine the specificity of the PCR amplification, the melting curve for Tm, its symmetry, and the lack of non-specific peaks were checked. All tests were conducted in triplicate. The expression ratio was recorded as the fold difference in quantity of real-time PCR product from samples. Each mRNA expression value was normalized against a housekeeping gene expression (GAPDH).

### Histological analysis

Tissue samples were fixed with 10% buffered formaldehyde, dehydrated and embedded in paraffin. Liver section (5 μm) was stained with haematoxylin and eosin (H&E) examined under light microscopy (Olympus CX31RBSF) to assess the hepatic changes.

The quantitative and semi-quantitative histological analysis was also used for scoring the histopathological variables by a veterinary pathologist. The mean numbers of marginated and infiltrated neutrophils were counted in the 10 random high power fields of the microscope. Thereafter, scoring between 0-4 was performed as follows: score 0 = 0 up to 9 neutrophils, score 1 = 10 up to 19 neutrophils, score 2 = 20 up to 29 neutrophils, score 3 = 30 up to 39 neutrophils, score 4 = more than 40 neutrophils. In addition, mononuclear cell infiltrations and Kupffer cell hyperplasia scorings were as follows: score 0 = normal condition, score 1 = the mild changes, score 2 = the average changes, score 3 = the severe changes, score 4 = more severe changes.

### Statistical analysis

Data are presented as Mean ± Standard Error (SE). The results were subjected to one-way ANOVA followed by Tukey’s HSD (Honestly Significant Differences) using SPSS 22.0 software. The significance was considered as *p* < 0.05.

## Results

### Essential oil analysis

Based on the GC/MS analyses, 21 known compounds were identified in the essential oil samples extracted from *Mentha longifolia* (Table 3). The major compounds were carvone (61.43%), limonene (27.77%), iso-dihydro carvone (3.47%) and caryophyllene (Isomer) (1.42%), respectively.

**Table 3.** Essential oil analysis prepared from *M. longifolia*.

No.	Compound	RI	%
1	α-Pinene	920.9677	0.87
2	β-Pinene	956.9892	0.81
3	Sabinene	953.2258	0.51
4	Myrcene	965.5914	0.33
5	Limonene	1003.226	27.77
6	Limonene oxide	1088.71	0.1
7	*iso*-Dihydrocarvone	1139.891	3.47
8	Carveol	1168.306	0.4
9	Pulegone	1173.224	0.81
10	Carvone	1188.525	61.43
11	Carvone oxide	1204.624	0.11
12	*iso*-Pulegone acetate	1239.306	0.12
13	*trans*-Carvyl acetate	1246.243	0.04
14	Carvyl acetate	1266.474	0.72
15	Bourbunene	1287.861	0.43
16	IsoCaryophyllene	1304.217	0.06
17	Caryophyllene (Isomer)	1315.663	1.42
18	Farnesene	1334.94	0.12
19	Caryophyllene (Isomer)	1341.566	0.14
20	Germacrene	1362.048	0.03
21	Caryophyllene epoxide	1442.038	0.31
	Total		100

### Free radical scavenging and antioxidant activities of M. longifolia essential oils

**Figure 1.**
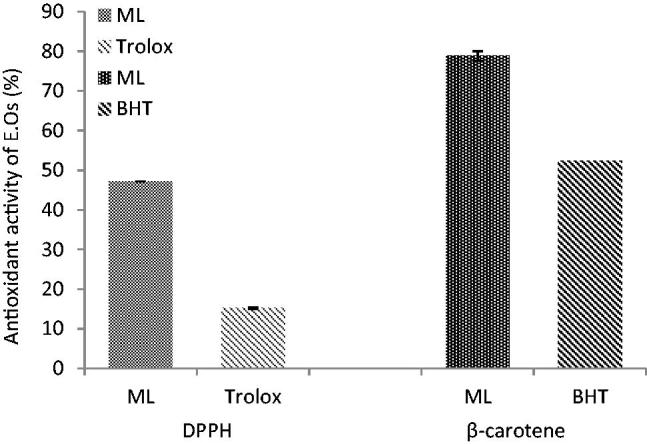
Free radical scavenging and antioxidant activities (%) of *M. longifolia* E.Os.

The antioxidative properties of essential oils analyzed by DPPH and β-carotene bleaching tests are presented in Figure 1. When compared to a standard antioxidant agent (trolox), it revealed that the essential oils from *M. longifolia* have strong radical scavenging activity. Addition of plant oils to the reaction mixture containing β-carotene and linoleic acid caused an inhibition in the formation of peroxidation products (Figure 1).

### The effect of *Mentha longifolia* essential oils on the oxidative stress parameters in septic rats

CLP operation caused a significant increase (*p* < 0.05) in LP as compared to the control group. The rats treated with essential oils at 50 and 100 mg/kg b.w doses significantly decreased the LP levels (*p* < 0.05). Also, the GSH and FRAP levels were significantly diminished 24 h after CLP as compared to the control group (*p* < 0.05). Treatment of rats with the obtained E.Os (50 and 100 mg/kg b.w) elevated both parameters to the normal values (*p* < 0.05). Moreover, indomethacin had partly the same effect as essential oils. In addition, the level of the detoxifying enzyme, GST, was not significantly changed in all groups as compared to the LAP group (*p* > 0.05) (Table 4).

**Table 4.** The effects of *M. longifolia* E.Os on the oxidative stress/antioxidant parameters and metabolizing enzymes in the septic rats.

Groups	LP (n mol/mg protein)	GSH (n mol/mg protein)	FRAP (µmol/L)	GST (n mol/min/mg protein)
LAP	10.34 ± 1.18	11.42 ± 1.1	407 ± 21.76	1126 ± 61.61
CLP	18.51 ± 1.53*	7.28 ± 0.67*	257 ± 10.98*	1173 ± 32.11
E.O 50	10.63 ± 0.84**	10.76 ± 0.96**	387 ± 9.3**	1258 ± 60.69
E.O 100	10.48 ± 0.93**	11 ± 0.89**	379 ± 12.86**	1513 ± 61.72
Indomethacin	11.8 ± 0.87**	11.26 ± 0.95**	280 ± 18.2	1076 ± 48.22

E.O: *M. longifolia* essential oils (50 & 100 mg kg b.w) treated groups.

* *p* < 0.05 is significantly considered between LAP and CLP group.

** *p* < 0.05 is significantly considered between CLP and treated groups. Data are presented as mean ± SD.

### The effect of *Mentha longifolia* essential oils on MPO activity

**Figure 2.**
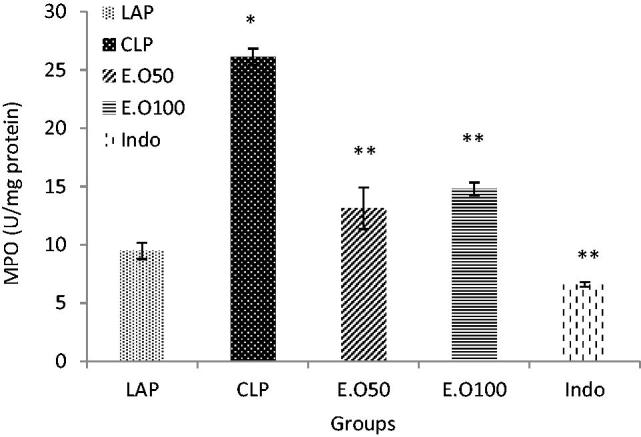
The effects of *M. longifolia* E.Os on MPO activity in the septic rats. E.O: *M. longifolia* essential oils (50 & 100 mg kg b.w) treated groups. *p < 0.05 is significantly considered between LAP and CLP group. **p < 0.05 is significantly considered between CLP and treated groups. Data are presented as mean ± SD.

There was a significant effect of CLP-induced sepsis on the liver MPO activity (Figure 2). Liver MPO activity increased significantly in the CLP group as compared to the control group (*p* < 0.05) (Figure 2). The rats treated with essential oils at 50 and 100 mg/kg b.w were capable of suppressing MPO activity in septic rats. The effects of essential oils on MPO were comparable to the effects of indomethacin as a positive group (Figure 2).

### The effect of *Mentha longifolia* essential oils on prostaglandin E2

**Figure 3.**
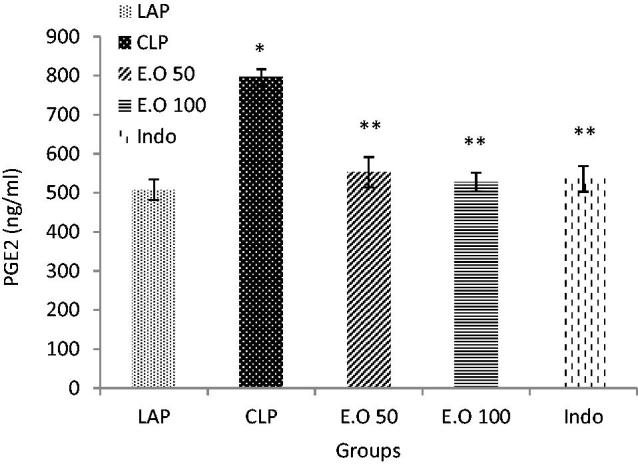
The effects of *M. longifolia* E.Os on prostaglandin E2 level in the septic rats. E.O: *M. longifolia* essential oils (50 & 100 mg kg b.w) treated groups. *p < 0.05 is significantly considered between LAP and CLP group. **p < 0.05 is significantly considered between CLP and treated groups. Data are presented as mean ± SD.

Prostaglandin E2 was increased in plasma after sepsis in relation to the LAP group (*p* < 0.05). Treatment of rats with the essential oils as well as indomethacin can return the levels of PGE2 to the normal rate (*p* < 0.05) (Figure 3).

### Effect of *Mentha longifolia* essential oils on the liver enzymes

As shown in Table 5, the serum levels of AST and ALT were drastically increased after sepsis (*p* < 0.05). E.Os and indomethacin treatments significantly decreased liver injury (*p* < 0.05) while, plasma ALP and total bilirubin were not affected in all the treated animals (*p* > 0.05) (Table 5).

**Table 5.** The effects of *M. longifolia* E.Os on the liver enzymes in the septic rats.

Groups	AST (u/l)	ALT (u/l)	ALP (u/l)	BILI (mg/dl)
LAP	132 ± 9.58	61 ± 5.35	364 ± 33.8	0.54 ± 0.05
CLP	317 ± 13.58*	136 ± 8.76*	400 ± 25.8	0.6 ± 0.05
E.O 50	154 ± 11.52**	75 ± 6.93**	360 ± 21.6	0.6 ± 0.04
E.O 100	160 ± 13.06**	75 ± 8.41**	362 ± 33.7	0.59 ± 0.04
Indomethacin	150 ± 11.72**	73 ± 4.48**	371 ± 30	0.54 ± 0.04

E.O: *M. longifolia* essential oils (50 & 100 mg.kg b.w) treated groups.

* *p* < 0.05 is significantly considered between LAP and CLP group.

** *p* < 0.05 is significantly considered between CLP and treated groups. Data are presented as mean ± SD.

### Relative gene expression results

**Figure 4.**
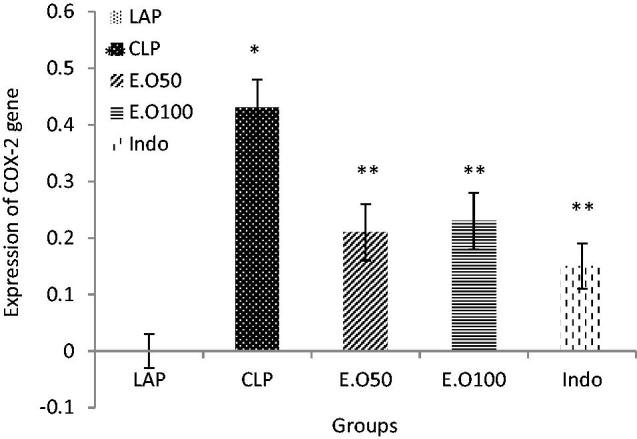
The effects of *M. longifolia* E.Os on COX-2 expression level in the septic rats. E.O: *M. longifolia* essential oils (50 & 100 mg kg b.w) treated groups. *p < 0.05 is significantly considered between LAP and CLP group. **p < 0.05 is significantly considered between CLP and treated groups. Data are presented as mean ± SD.

To investigate whether *M. longifolia* E.Os could regulate COX-2 expression, we used real-time PCR to measure the COX-2 mRNA expression after CLP operation. The distributions of mRNA expression level of COX-2 were normalized by GAPDH as shown in the Figure 4. In comparison to the LAP group (Mean 0 ± 0 SD), the COX-2 expression level was increased in CLP group (0.43 ± 0.05) (*p* < 0.05), which was significantly reduced by *M. longifolia* E.Os at two doses (0.21 ± 0.05, 0.23 ± 0.05) (*p* < 0.05). Also, indomethacin as an NSAID drug could decrease the COX-2 expression (0.15 ± 0.11) as well (*p* < 0.05). In other words, no significant difference was found between the essential oil and indomethacin groups rather than difference between treatment groups with septic group (*p* < 0.05) (Figure 4).

### Histological findings

**Figure 5.**
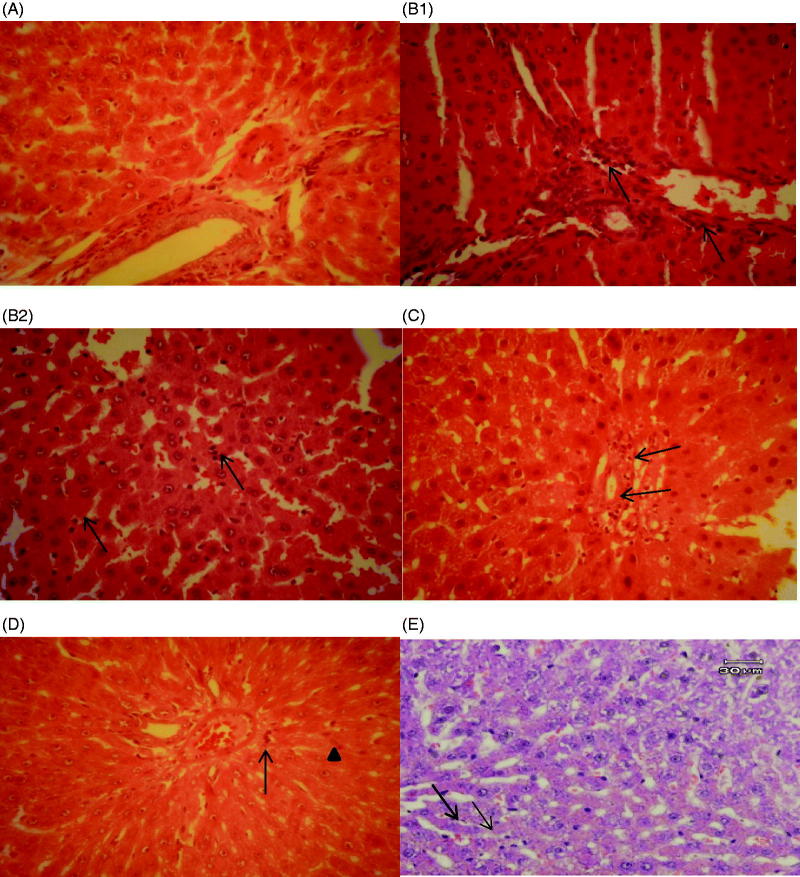
Histopathological studies. (A) LAP group, the portal tract and the hepatocytes in normal condition. (B1) CLP group, neutrophil infiltration in the portal tract (arrows). (B2) CLP group, neutrophil infiltration in the sinusoids which can be seen easily with their dark nuclei (arrows). (C) E.O50, mild infiltration of neutrophils in the portal tract (arrows). H&E, 400*. (D) E.O100 group, reduced neutrophil infiltration (arrow head) and a few Kupffer cells (thin arrow) could be seen in the picture. H&E, 400*. (E) Indomethacin group, a few infiltrated neutrophils (arrows) could be seen in the picture. H&E, 400*.

Histopathologic assessment of liver specimens revealed that there were some mild changes *viz.* congestion and granular degeneration of the hepatocytes in the LAP group (Figure 5(A)). In the CLP group, severe congestion, interstitial oedema and even margination of neutrophils in the venules and sinusoids were observed. Neutrophils and mononuclear cells were also infiltrated in the portal tracts and sinusoids in the septic group. Kupffer cell hyperplasia and granular degeneration were the other observed changes in the CLP group. There weren’t any signs of necrosis in hepatocytes. All the changes in the CLP group revealed a kind of hepatitis called Non Specific Reactive Hepatitis (Figure 5(B1,B2)). Surprisingly, the essential oil treated groups improved the histopathological lesions. There was mild infiltration of neutrophils in the portal tract in the E.O50 treated group in comparison with the CLP group (Figure 5(C)). In addition, a few infiltrated neutrophils and normal amount of Kupffer cells were observed in E.O100 and indomethacin treated groups (Figure 5(D,E)).

As shown in Table 6, the CLP group obviously showed the neutrophil margination and infiltration, mononuclear cell infiltration and Kupffer cell hyperplasia as compared with the LAP group (*p* ≤ 0.05). Concerning portal inflammation, it was also meaningful in the CLP group in comparison with the LAP group (*p* ≤ 0.05). However, there weren’t obvious differences regarding granular degeneration and inflammatory foci between all study groups (*p* > 0.05). To confirm the results seen in Figure 5, all the treatment groups prominently reduced neutrophil margination and infiltration, mononuclear cells infiltration, Kupffer cell hyperplasia and portal inflammation in comparing with the CLP group (*p* ≤ 0.05).

**Table 6.** Mean values and standard error of histopathologic variables of the liver specimens in the study groups.

Study groups	Neutrophil margination and infiltration	Granular degeneration	Inflammatory foci	Mononuclear cell infiltration & Kupffer cell hyperplasia	Portal inflammation
LAP	0 ± 0	0.4 ± 0.24	0 ± 0	0 ± 0	0 ± 0
CLP	2.75 ± 0.25*	0.75 ± 0.75	1.5 ± 0.86	3 ± 0.4*	2.25 ± 0.25*
E.O50	1.2 ± 0.37**	0 ± 0	0 ± 0	1.4 ± 0.24**	0.8 ± 0.48**
E.O100	1.16 ± 0.16**	0.33 ± 0.21	0.66 ± 0.49	1.5 ± 0.22**	0.66 ± 0.21**
Indomethacin	0.5 ± 0.28**	0 ± 0	0 ± 0	1 ± 0**	0.25 ± 0.25**

E.O: *M. longifolia* essential oils (50 & 100 mg.kg b.w) treated groups.

* : Having significant differences in comparison with the LAP group.

** : Having significant differences in comparison to the CLP group.

## Discussion

Sepsis can be caused by bacteria infections wherein protein mediators play a critical role in mobilizing the body’s inflammatory response to contain and eradicate microbial infections. Inflammation causes tissue injury and dysfunction of vital organs. The excessive production of reactive oxygen species (ROS), associated with inflammation, leads to an oxidative stress state which is an important contributing factor to the high mortality rates associated with several diseases (Victor et al. 2004; Prasad et al. 2017).

This study originality elucidates the *in vivo* antioxidant and anti-inflammatory potential of Iranian *M. longifolia* essential oils through sepsis. The oils possessed carvone and limonene as major compounds concomitant with potential *in vitro* antioxidative properties measured through DPPH and beta-carotene bleaching tests (Table 3 and Figure 1). Many studies showed that carvone and limonene in *Mentha* species exhibited antimicrobial, antifungal and antioxidant activities (Aggarwal et al. 2002; Sartoratto et al. 2004; Gulluce et al. 2007; Kamkar et al. 2009; Singh et al. 2011; Zare Bidaki et al. 2015). Based on the previous studies, we expected that administration of oils to a septic animal can interfere in parameters associated with antioxidant/oxidative stress reactions.

The data indicated that *M. longifolia* E.Os could modulate antioxidant defense system by interfering in the antioxidant/oxidative stress parameters such as LP, GSH, FRAP and MPO during CLP development (Table 4, Figure 2). Increased intraluminal levels of MPO is a hallmark of systemic inflammatory disease and are viewed as primary host defense mechanism during sepsis, pneumonia, and other pathogen-related diseases (Witko-Sarsat et al. 2000) resulting to the increased lipid peroxidation (LP) *via* oxidative stress invasion (Blokhina et al. 2003). Several studies revealed that reactive intermediate(s) serves as the relevant participant(s) in MPO initiated lipid peroxidation *in vivo*. In a recent study, Zhang et al. (2002) employed a systematic approach to isolate and chemically define the low molecular weight components in plasma capable of enabling MPO to initiate peroxidation of plasma lipids leading to MDA production which has been used as a marker for tissue damage.

Glutathione (GSH) and glutathione S-transferase are among the major antioxidant defense systems. Cytosolic glutathione S-transferases (GSTs) are a complex multigene family of enzymes that possess many biological functions, the most important of which is detoxification of a range of xenobiotic compounds by their metabolite conjugation produced by CYP450 to GSH (Oakley 2011; Satheesh et al. 2011). In addition, GSH plays an important role in the maintenance of protein and lipid integrity, and provides major protection in oxidative injury against oxidative damage (Narasimhan et al. 2012). The results of the present study are in concurrence with the reports of the following two studies (Bacanli et al. 2014; Taner et al. 2014), supporting the idea that GSH decrease after CLP-induced liver injury (Table 4), is one of the important factors that permit lipid peroxidation and subsequent tissue damage. The rise in MDA level concomitant with diminished GSH level (Table 4) indicated the role of oxidative mechanism in sepsis-induced tissue damage (Şener et al. 2005; Kagan Coskun et al. 2011). Accordingly, no significant alteration in the GST activity (Table 4) indicated no probable effective role of this enzyme in the detoxification of septic rat.

On the other hand, FRAP levels, as a factor in oxidative stress/antioxidant balancing (Dadkhah et al. 2006), was needed by the essential oils as an antioxidant to protect the liver against sepsis damages. Decrease of FRAP in plasma of CLP-treated rats (Table 4) may be due to enzymatic and non-enzymatic antioxidant activities causing an increased resistance and/or decreased susceptibility of the liver to free radical attack (Dadkhah et al. 2006).

Plant derived compounds have long been used as natural resources of traditional remedies (Adas et al. 2009). Several studies showed that plant products are very useful in treating liver diseases. Essential oils or extracts from the plants could be as effective and reliable hepatoprotective agents (Thyagarajan et al. 2002; Fatma et al. 2016). Furthermore, with respect to the increasing side effects of antibiotics and non-steroidal anti-inflammatory drugs in sepsis treatment, medicinal plants with antibacterial and antioxidant activities could be suitable alternative treatments.

The present study results indicated that *M. longifolia* E.Os at 50 and 100 mg/kg b.w could significantly (*p* < 0.05) reverse the hepatic cellular GSH, LP, FRAP and MPO levels (Table 4, Figure 2). The effect of essential oils on MPO, GSH, LP and FRAP was comparable to the effects of indomethacin group (Table 4, Figure 2). Our study signifies that the hepatoprotective activity of *M. longifolia* may be due to its antioxidant activity, resulting from the presence of some antioxidant compounds in the essential oil. Fatemi et al. (2010) represented the effect of γ-irradiated caraway essential oils in experimental sepsis. Their study implied that the caraway essential oils enriched with carvone and limonene compounds can improve the liver injury by modulating oxidative stress parameters such as LP, MPO and GSH. Bacanli et al. (2016) showed that rosmarinic acid treatment has protective role against sepsis-induced oxidative damage in Wistar Albino rats.

In addition, the essential oil could return the COX-2 expression rate to the LAP group (Figure 4). COX-2 expression leads to elevated protein production which in turn causes inflammation (Esmaeili et al. 2011). Also, COX is an enzyme that mediates the bioconversion of arachidonic acid to inflammatory prostaglandins. An increase of PGE2 in CLP groups (Figure 3), can be due to COX-2 over expression, and is considered as the most important downstream effector of COX-2.

It is clear that gene transcription is controlled by several transcription factors which in turn control the production of one protein (Cooper 2000). As confirmed in the present study, the probable anti-inflammatory and antioxidant agents of *M. longifolia* E.O can decrease the expression of inflammatory mediators leading to decreased expression of COX-2 in parallel with diminished PGE2 level (Figures 3 and 4). Likewise, indomethacin as non-steroidal anti-inflammatory drugs (NSAIDs) is the competitive inhibitors of cyclooxygenase (COX) that could affect related modulatory parameters (Tables 4 and 5; Figures 2–4). Recent studies indicated that *N*-acetylcysteine decreased lipid peroxidation and reduced the expression of several inflammatory mediators in a rat model of lung injury (Cuzzocrea et al. 2001). *In vivo* anti-inflammatory effects of some solvent extracts of *Mentha spicata* L. (Arumugam et al. 2008) were reported. Recently, it was documented that baicalein treatment provides protection against the CLP-induced liver injury *via* inhibition of inflammatory response and reduction of hepatic apoptosis (Liu et al. 2015).

The existences of hepatic enzymes such as AST and ALT in the cytosol were naturally correlated with liver damage in the blood stream. So, their estimation in the serum is a useful quantitative marker of the extent and type of hepatocellular damage (Rasooli et al. 2016). *Mentha longifolia* E.Os in addition to indomethacin could improve liver damage induced by sepsis. E.Os in both doses (50 & 100 mg/kg b.w) could decrease serum activities of ALT, AST enzymes to the normal rate (Table 5). Similarly, results of the study by Rašković et al. (2014) showed that rosemary essential oil could restore the elevated ALT and AST activities. Also, it was reported that sesame oil affected oxidative stress and hepatic injury after cecal ligation and puncture in rats (Hsu et al. 2004, 2006). Serum bilirubin is one of the true tests of liver functions since it reflects the ability of the liver to take up and process bilirubin into bile (Sabina et al. 2013). However, ALP and total bilirubin levels were not significantly (*p* > 0.05) changed in the CLP group as compared to the LAP group (Table 5) indicating no biliary damage. These results were confirmed by pathological examination on liver indicating that essential oils from *M. longifolia* plus indomethacin can improve the liver injuries in sepsis induced by CLP model (Figure 5).

## Conclusions

This study demonstrated that antioxidative *M. longifolia* E.Os administration converted sepsis-induced oxidative injury by positively affecting COX-2 expression leading to decreased prostaglandin E2 level, as effective as indomethacin. This positive effect was seen to be reflected in alleviating oxidative stress/antioxidant status *viz*. MPO, LP, GSH and FRAP in sepsis. This may serve *M. longifolia* E.Os as a potential therapeutic agent for liver injury in sepsis.
